# In silico design of a *Zika virus* non-structural protein 5 aiming vaccine protection against zika and dengue in different human populations

**DOI:** 10.1186/s12929-017-0395-z

**Published:** 2017-11-23

**Authors:** Lorrany dos Santos Franco, Paloma Oliveira Vidal, Jaime Henrique Amorim

**Affiliations:** Laboratório de Microbiologia, Centro das Ciências Biológicas e da Saúde, Universidade Federal do Oeste da Bahia, Rua Bertioga, 892, Morada Nobre II, Barreiras, Bahia CEP 47810-059 Brazil

**Keywords:** *Zika virus*, *Dengue virus*, Vaccine, Epitopes, Bioinformatics

## Abstract

**Background:**

The arboviruses *Zika virus* (ZIKV) and *Dengue virus* (DENV) have important epidemiological impact in Brazil and other tropical regions of the world. Recently, it was shown that previous humoral immunity to DENV enhances ZIKV replication *in vitro*, which may lead to more severe forms of the disease. Thus, traditional approaches of vaccine development aiming to control viral infection through neutralizing antibodies may induce cross-reactive enhancing antibodies. In contrast, cellular immune response was shown to be capable of controlling DENV infection independently of antibodies. The aim of the present study was to design a flavivirus NS5 protein capable of inducing a cellular immune response against DENV and ZIKV.

**Methods:**

A consensus sequence of ZIKV NS5 protein was designed among isolates from various continents. Epitopes were predicted for the most prevalent alleles of class I and II HLA in the Brazilian population. Then, this epitopes were analyzed with regard to their conservation, population coverage and distribution along the whole antigen.

**Results:**

Nineteen epitopes predicted to be more reactive (percentile rank <1) and 100% conserved among ZIKV and DENV serotypes were selected. The distribution of such epitopes along the protein was shown on a three-dimensional model and population coverage was calculated for different regions of the world. The designed protein was predicted to be stable and the distribution of selected epitopes was shown to be homogeneous along domains. The population coverage of selected epitopes was higher than 50% for most of tropical areas of the world.

**Conclusion:**

Such results indicate that the proposed antigen has the potential to induce protective cellular immune response to ZIKV and DENV in different human populations of the world.

**Electronic supplementary material:**

The online version of this article (10.1186/s12929-017-0395-z) contains supplementary material, which is available to authorized users.

## Background

The Flavivirus genus of the *Flaviviridae* family consists of more than 70 viruses, many of which are arthropod-borne viruses, i.e. arboviruses [1]. It is estimated that more than 390 million people are infected annually by any of the four serotypes of *Dengue virus* (DENV1–4) [2], the most prevalent of the emerging arboviruses. Recently, *Zika virus* (ZIKV), an emergent pathogen previously associated with mild infections, started to be associated with microcephaly in babies and *Guillain-Barré* syndrome in the Americas [3–5]. Together, these two Flavivirus consist a world public health concern for which there is not specific treatment. For now, controlling arthropod vectors is the most effective and available method to prevent DENV and ZIKV burden. However, effective vaccines are needed in order to complement mosquito control programs, once that eradication of vectors is challenging and time consuming.

The only anti-DENV vaccine candidate approved for use in humans is based on a chimera between DENV and *Yellow fever virus* (YFV) [6–10]. It consists of chimeric viruses for each of DENV serotypes in which genomic sequences coding for YFV envelope proteins were replaced by those of DENV. Thus, specific immunity against DENV is concentrated on antigens that mainly induce generation of antibodies. Unfortunately, especially for DENV2, the vaccine formulation based on those chimeric viruses did not achieve the expected protective efficacy in phase III clinical trials in different regions of the world. In addition, it was reported to present higher incidence of hospitalization for dengue in year 3 after vaccination among children younger than 9 years of age [10]. Some severe forms of dengue are mediated by a phenomenon called antibody-dependent enhancement, in which immunoglobulins produced in response to a previous DENV serotype cross-react with viral particles of a second serotype and mediate enhanced infection of Fc-γ receptor bearing cells. Such enhanced infection leads to higher viral loads and severe disease. Relevantly, such phenomenon was reported between DENV and ZIKV [11–14]. Thus, the risk of inducing antibody-mediated enhancement of infection between these two major arboviruses will depend on the profile of immune response induced.

In contrast to envelope proteins, which are structural proteins, non-structural proteins are not present in viral particles and are not able to contribute directly to antibody-dependent enhancement. In addition, they were shown to be major targets for CD4^+^ and CD8^+^ T lymphocytes involved with control of DENV spread and intracellular replication [15–17]. Also, CD8^+^ T lymphocytes with multifunctional cytokine secretion patterns were found in volunteers immunized with a live attenuated tetravalent dengue vaccine [18]. Such lymphocytes target highly conserved epitopes located on non-structural proteins and this immunological pattern fits with those found after natural infections in which control of disease was observed [18]. Moreover, our last reports showed that protection against DENV is achieved independently of humoral immunity [19]. CD4^+^ and CD8^+^ T lymphocytes targeting non-structural proteins, mainly NS5, were shown to be essential for protection capacity. When a recombinant purified form of NS5 protein was used as a vaccine antigen, we achieved 70% of protection working on a mouse model [20].

The NS5 protein is a well-conserved antigen between DENV and ZIKV. It is the major DENV target for cellular immune response. In this study we aimed to design a vaccine antigen capable of inducing cross-protective cellular immunity against DENV and ZIKV. Here, we predicted NS5 common epitopes for DENV and ZIKV to be presented by HLA (human leucocyte antigen) encoded by alleles of different populations of the world. The designed antigen was shown to present a homogeneous distribution of epitopes and its predicted 3D model was shown to fit with the structure of a ZIKV native NS5. The population coverage of the antigen was higher than 50% for most of tropical regions of the world. Thus, it is possible to predict vaccine efficacy by region. In summary, we present a designed antigen which may be a valuable alternative in order to control the burden of DENV and ZIKV.

## Methods

### NS5 sequences database building

One database was built with NS5 protein amino acid sequences from ZIKV and DENV isolated in different continents of the world. Sequences in FASTA format were retrieved from the National Center for Biotechnology Information (NCBI) protein database (http://www.ncbi.nlm.nih.gov/protein/). Criteria for selecting sequences were: i) complete annotation of the NS5 protein and ii) absence of undefined amino acid in the sequence. The database consisted of 153 NS5 protein amino acid sequences from the four serotypes of DENV and of 41 NS5 protein amino acid sequences ZIKV isolates. A sequence of NS5 protein from *Spondwedi virus* (accession number: ABI54480.1) was included to serve as a control. Accession numbers of DENV and ZIKV NS5 sequences are shown in Additional file 1: Tables S1 to S5.

### Multiple alignment, consensus sequence design and phylogeny of the ZIKV sequences

Evolutionary analyses were conducted in MEGA7. Multiple Alignments of DENV and ZIKV sequences were carried out using the ClustalW method [21]. A NS5 consensus sequence among ZIKV isolates was designed based on results obtained from multiple alignment of ZIKV NS5 proteins, using MegAlign program from Lasergene. The evolutionary history of DENV and ZIKV NS5 proteins was inferred using the Neighbor-Joining method. *Spondwedi virus* was used as a control. A bootstrap test of 1000 replicates was applied. The evolutionary distances were computed using the Poisson correction method. The analysis involved 195 amino acid sequences.

### Survey of the most frequent HLA alleles in the Brazilian population

The allelic frequency of both, class I and class II HLA, in the Brazilian population, was retrieved from NCBI database (https://www.ncbi.nlm.nih.gov/projects/gv/mhc/ihwg.cgi). Alleles frequent in at least 3% of the Brazilian population were selected.

### Epitope prediction analysis

Epitopes within the NS5 protein consensus were predicted using the IEDB (Immune epitope database) analysis resource (http://tools.immuneepitope.org/mhci/), IEBD recommended method. NetMHCpan method was also used when prediction was not possible by using IEBD recommended method. The epitopes from the NS5 consensus were ranked by their percentile rank value and those with a percentile rank ≤1 were selected. Epitopes with 8, 9, 10 and 11 amino acids in length were considered for Class I HLA and 15 amino acids for Class II HLA. A file containing the best ranked epitopes was created.

### Analysis of conservancy of predicted epitopes

The IEDB conservancy analysis tool (http://tools.iedb.org/conservancy) was used to determine the conservancy of the predicted epitopes among ZIKV lineages and DENV serotypes. Only predicted epitopes with a percentile rank ≤1 were used in this analysis. Only epitopes 100% conserved among all sequences of DENV and ZIKV were selected.

### Population coverage analyses

Epitopes previously selected were used to determine the population coverage by the IEDB population coverage calculation tool (http://tools.immuneepitope.org/tools/population/iedb_input). All of the selected epitopes were analyzed for similarity with human proteome using BLAST program (http://www.ncbi.nlm.nih.gov/BLAST/) to verify if they would not trigger autoimmunity.

### Structural biology analysis

The ZIKV NS5 consensus amino acid sequence was used to compute a 3D model using the I-TASSER modeling method. The best computed model was subjected to TM-align structural alignment program to match the first I-TASSER model to all structures in the PDB library (Protein Data Bank - http://www.rcsb.org). The top 1 model was also used to determine epitope distribution in protein domains. Epitopes were marked in the NS5 protein 3D model using the PyMOL program aiming to access their distribution.

## Results

### ZIKV NS5 phylogeny

**Fig. 1:**
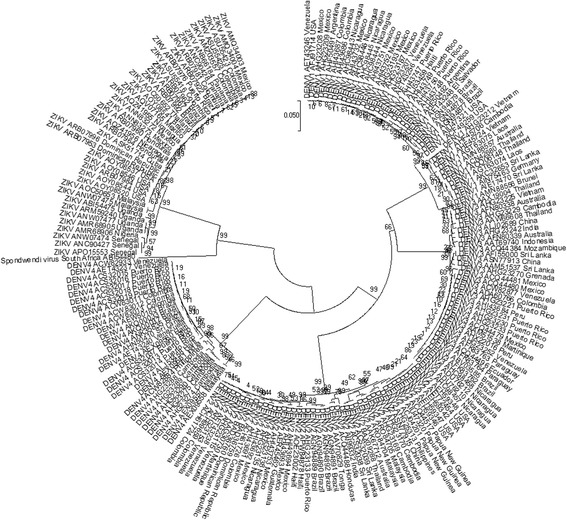
Evolutionary history of selected amino acid sequences of ZIKV and DENV NS5 proteins. The tree shows that NS5 sequences from Brazilian ZIKV isolates grouped with those from Asian ZIKV lineage. The evolutionary history was inferred by using the Maximum Likelihood method based on the JTT matrix-based model [1]. The percentage of trees in which the associated taxa clustered together is shown next to the branches. Initial tree(s) for the heuristic search were obtained automatically by applying Neighbor-Join and BioNJ algorithms to a matrix of pairwise distances estimated using a JTT model, and then selecting the topology with superior log likelihood value. The tree is drawn to scale, with branch lengths measured in the number of substitutions per site. The analysis involved 195 amino acid sequences. All positions containing gaps and missing data were eliminated. There were a total of 896 positions in the final dataset. Evolutionary analyses were conducted in MEGA7

In order to verify the origin of ZIKV isolates from Brazil and the Americas we carried out a phylogenetic analysis with NS5 amino acid sequences from ZIKV from throughout the world. We also included NS5 sequences from DENV serotypes from different genotypes isolated in different continents in order to verify similarities among ZIKV and DENV proteins. A comprehensive phylogenetic analysis of NS5 amino acid sequences based on world representative strains of ZIKV and DENV is shown in Fig. 1. NS5 sequences from Brazilian and American isolates grouped with those from the Asian lineage of ZIKV. None of the NS5 sequences from American isolates grouped with those from the African lineage of ZIKV. This result indicates that Brazilian and American ZIKV isolates analyzed in this study belong to the Asian lineage. In addition, the phylogeny indicates that both, DENV and ZIKV NS5 proteins diverged before their separation into two different viral species. In other words: proteins diverge since the root of the phylogenetic tree.

### Consensus sequence design

**Fig. 2:**
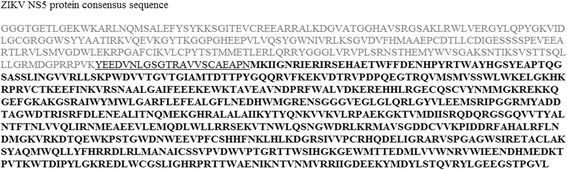
Consensus sequence of the ZIKV NS5 protein. Amino acid sequences from ZIKV NS5 database were used to construct a consensus sequence. The methyltransferase MTase domain is shown in gray, the linker region between MTase and RdRp domains is shown in black and underlined and the RdRp domain is shown black and bold

Although it has been shown that Brazilian and American ZIKV isolates belong to the Asian lineage, all the sequences contained in ZIKV database were used in the design of the consensus. It means that African sequences were also used. The option for including them was based on the aim of preventing disease with imported viruses from African lineage. Attempts to design a consensus NS5 protein between ZIKV and DENV resulted in a non-stable 3D model (data not shown). The ZIKV NS5 consensus is shown in Fig. 2.

### The most frequent alleles in Brazilian population were selected

The survey of HLA frequencies in Brazilian population demonstrated that the most frequent are those in Table 1. Eleven alleles were selected for HLA-A: A*01:01, A*02:01, A*02:02, A*02:24, A*03:01, A*11:01, A*24:02, A*29:02, A*31:01, A*33:03 e A*68:02. Seven alleles were selected for HLA-B: B*07:02, B*08:01, B*08:05, B*18:01, B*35:01, B*44:03, B*51:01. Eleven alleles were selected for HLA-C: C*02:02, C*03:03, C*03:04, C*04:01, C*05:01, C*07:01, C*07:02, C*08:02, C*12:03, C*15:02, C*17:01 and thirteen for HLA-DRB1 (DRB1*01:01, DRB1*01:02, DRB1*03:01, DRB1*04:01, DRB1*07:01, DRB1*08:04, DRB1*11:01, DRB1*11:04, DRB1*13:01, DRB1*13:02, DRB1*14:01, DRB1*15:01, DRB1*15:03).

**Table 1:** Frequency of class I and class II HLA alelles (≥ 3%) in the Brazilian population

Class	*Loci*	Alelle	Frequence
Class I	HLA-A	A*01:01	0.091
		A*02:01	0.192
		A*02:02	0.040
		A*02:24	0.040
		A*03:01	0.051
		A*11:01	0.061
		A*24:02	0.081
		A*29:02	0.030
		A*31:01	0.040
		A*33:03	0.030
		A*68:02	0.035
	HLA-B	B*07:02	0.101
		B*08:01	0.043
		B*08:05	0.036
		B*18:01	0.051
		B*35:01	0.051
		B*44:03	0.035
		B*51:01	0.051
	HLA-C	C*02:02	0.033
		C*03:03	0.048
		C*03:04	0.048
		C*04:01	0.138
		C*05:01	0.062
		C*07:01	0.157
		C*07:02	0.081
		C*08:02	0.076
		C*12:03	0.038
		C*15:02	0.033
		C*17:01	0.033
Class II	HLA-DRB1	DRB1*01:01	0.055
		DRB1*01:02	0.035
		DRB1*03:01	0.121
		DRB1*04:01	0.035
		DRB1*07:01	0.075
		DRB1*08:04	0.030
		DRB1*11:01	0.060
		DRB1*11:04	0.035
		DRB1*13:01	0.070
		DRB1*13:02	0.050
		DRB1*14:01	0.035
		DRB1*15:01	0.050
		DRB1*15:03	0.045

### Epitope prediction and conservation analyses

Epitopes with higher binding affinity to the HLA molecules selected before were identified. Then, they were analyzed with regard to their conservation in all the ZIKV NS5 sequences contained in the database previously prepared. Epitopes with percentile rank ≤1 (high binding affinity) and 100% conserved among ZIKV and DENV NS5 sequences were selected. We selected 19 epitopes (Table 2) 100% conserved among all DENV and ZIKV NS5 amino acid sequences analyzed in this study. This result indicates that NS5 protein concentrates a relevant number of epitopes highly conserved among ZIKV lineages and DENV serotypes. In addition, conservation analyses also showed that 624 epitopes are 100% conserved among ZIKV lineages, 85 epitopes are 100% conserved among ZIKV and DEN1, 102 epitopes are 100% conserved among ZIKV and DEN2, 61 epitopes are 100% conserved among ZIKV and DEN3 and 126 epitopes are 100% conserved among ZIKV and DEN4. This result indicates that the consensus ZIKV NS5 protein share different numbers of epitopes with each of your homologous proteins.

**Table 2:** ZIKV NS5 predicted epitopes 100% conserved among ZIKV lineages and DENV serotypes and their respective population coverage rates (%) considering the Brazilian population

Epitope	Location^a^	Alleles	Population coverage
LSRNSTHEMY	211–220	HLA-A*01:01	13.87%
CVYNMMGKR	451–459	HLA-A*03:01, HLA-A*31:01, HLA-A*33:03	12.06%
CVYNMMGKREK	451–461	HLA-A*03:01, HLA-A*11:01	12.06%
AIWYMWLGAR	474–483	HLA-A*31:01	11.06%
WYMWLGAR	476–483	HLA-A*31:01, HLA-A*33:03, HLA-C*04:01	33.73%
RAIWYMWLGAR	473–483	HLA-A*33:03	3.9%
IWYMWLGAR	475–483	HLA-A*31:01, HLA-A*33:03	11.06%
LEFEALGF	485–492	HLA-B*18:01, HLA-C*04:01	8.0%
YADDTAGW	532–539	HLA-C*03:03, HLA-C*05:01, HLA-C*08:02, HLA-C*17:01	9.64%
RAIWYMWL	473–480	HLA-C*03:03, HLA-C*07:02, HLA-C*15:02	9.64%
YADDTAGWDT	532–541	HLA-C*05:01, HLA-C*08:02	9.7%
YADDTAGWDTR	532–542	HLA-C*05:01, HLA-C*08:02	9.7%
YADDTAGWD	532–540	HLA-C*05:01, HLA-C*12:03	9.7%
ADDTAGWD	533–540	HLA-C*05:01	9.7%
GRGGWSYY	83–90	HLA-C*07:01, HLA-C*07:02	23.83%
SRNSTHEM	212–219	HLA-C*07:01, HLA-C*07:02	23.83%
SRNSTHEMY	212–220	HLA-C*07:01, HLA-C*07:02	23.83%
SRNSTHEMYW	212–221	HLA-C*07:01	23.83%
SRAIWYMWL	472–480	HLA-C*07:02	19.15%

^a^Location of the predicted epitope into the ZIKV NS5 consensus amino acid sequence

### Population coverage of epitopes predicted in ZIKV NS5 consensus vary in different human populations of the planet

As shown in conservancy analyses, we selected nineteen epitopes with 100% identity among all the ZIKV and DENV NS5 sequences from databases. Such epitopes were shown to present a relevant population coverage with regard to the most prevalent HLA alleles in the Brazilian population, ranging from 3.9% to 33.73% (Table 2). Together, they presented accumulated population coverages of 74.07% and 68.70% for Brazilian and United States populations, respectively. In the Brazilian population, coverages found by ethnicity were 66.07% for Amerindian, 46.71% for Caucasoid, and 75.63% for Mixed. In the population of United States, coverages found by ethnicity were 50.64% for Amerindian, 64.25% for Asian, 0.0% for Austronesian, 64.12% for Black, 85.29% for Caucasoid, 71.07% for Hispanic, 72.82% for Mestizo and 70.45% for Polynesian. In addition, such epitopes presented population coverages higher than 50% for different continents and subcontinents of the world: North America, South America, Europe, West Africa, Central Africa, North Africa, South Africa, Northeast Asia, South Asia, Eats Asia, Southeast Asia, Southwest Asia and Oceania. Relevantly, selected epitopes contained in the consensus ZIKV NS5 presented population coverages of 81.76%, 68.5%, 64.63%, 65.32% and 59.37% for Europe, North America, South America, East Asia and Southeast Asia, respectively (Table 3). These results indicate that the consensus NS5 protein proposed in this study is a promising antigen with regard to induce cellular immune response against ZIKV and DENV in different human populations of the world.

**Table 3:** Total population coverage of selected epitopes for different regions of the world

Epitope	North America	Central America	South America	Europe	East Africa	West Africa	Central Africa	North Africa	South Africa	Northeast Asia	South Asia	East Asia	Southeast Asia	Southwest Asia	Oceania
LSRNSTHEMY	12.72%	0.00%	6.03%	25.67%	11.24%	7.31%	2.84%	14.18%	0.22%	3.44%	13.80%	2.55%	2.10%	14.66%	11.38%
CVYNMMGKR	13.11%	0.00%	6.37%	25.05%	7.49%	9.48%	12.29%	8.96%	0.14%	3.05%	12.33%	1.47%	1.18%	9.15%	4.04%
CVYNMMGKREK	13.11%	0.00%	6.37%	25.05%	7.49%	9.48%	12.29%	8.96%	0.14%	3.05%	12.33%	1.47%	1.18%	9.15%	4.04%
AIWYMWLGAR	7.09%	0.00%	16.56%	4.72%	2.11%	1.71%	1.14%	3.40%	0.07%	4.08%	8.42%	13.92%	2.65%	5.82%	3.28%
WYMWLGAR	7.09%	0.00%	16.56%	4.72%	2.11%	1.71%	1.14%	3.40%	0.07%	4.08%	8.42%	13.92%	2.65%	5.82%	3.28%
RAIWYMWLGAR	6.62%	0.00%	2.55%	0.91%	2.34%	12.54%	7.21%	6.08%	0.07%	11.17%	15.47%	16.92%	16.79%	5.21%	0.64%
IWYMWLGAR	7.09%	0.00%	16.56%	4.72%	2.11%	1.71%	1.14%	3.40%	0.07%	4.08%	8.42%	13.92%	2.65%	5.82%	3.28%
LEFEALGF	6.11%	0.00%	4.86%	11.8%	8.12%	4.85%	3.75%	5.85%	0.06%	0.68%	2.51%	0.0%	4.64%	5.91%	1.22%
YADDTAGW	6.48%	0.00%	4.03%	8.6%	1.72%	0.32%	0.9%	0.99%	0.13%	9.52%	2.98%	20.72%	7.96%	1.75%	12.2%
RAIWYMWL	6.48%	0.00%	4.03%	8.6%	1.72%	0.32%	0.9%	0.99%	0.13%	9.52%	2.98%	20.72%	7.96%	1.75%	12.2%
YADDTAGWDT	7.51%	0.00%	6.38%	10.3%	2.6%	2.6%	2.62%	5.92%	0.00%	0.93%	4.09%	1.06%	0.15%	3.5%	5.44%
YADDTAGWDTR	7.51%	0.00%	6.38%	10.3%	2.6%	2.6%	2.62%	5.92%	0.00%	0.93%	4.09%	1.06%	0.15%	3.5%	5.44%
YADDTAGWD	7.51%	0.00%	6.38%	10.3%	2.6%	2.6%	2.62%	5.92%	0.00%	0.93%	4.09%	1.06%	0.15%	3.5%	5.44%
ADDTAGWD	7.51%	0.00%	6.38%	10.30%	2.60%	2.60%	2.62%	5.92%	0.00%	0.93%	4.09%	1.06%	0.15%	3.50%	5.44%
GRGGWSYY	16.98%	0.00%	13.05%	25.11%	21.52%	21.54%	23.17%	24.53%	0.39%	1.53%	11.44%	11.64%	7.22%	11.85%	4.22%
SRNSTHEM	16.98%	0.00%	13.05%	25.11%	21.52%	21.54%	23.17%	24.53%	0.39%	1.53%	11.44%	11.64%	7.22%	11.85%	4.22%
SRNSTHEMY	16.98%	0.00%	13.05%	25.11%	21.52%	21.54%	23.17%	24.53%	0.39%	1.53%	11.44%	11.64%	7.22%	11.85%	4.22%
SRNSTHEMYW	16.98%	0.00%	13.05%	25.11%	21.52%	21.54%	23.17%	24.53%	25.18%	1.53%	11.44%	11.64%	7.22%	11.85%	4.22%
SRAIWYMWL	21.28%	0.00%	28.04%	22.28%	6.29%	4.16%	12.72%	7.26%	0.28%	28.5%	16.05%	20.09%	33.1%	12.49%	16.87%
Total	68.5%	0.00%	64.63%	81.76%	50.55%	51.39%	53.14%	58.57%	1.0%	51.69%	63.85%	65.32%	59.37%	54.4%	48.67%

### 3D NS5 model and epitope distribution on the protein

**Fig. 3:**
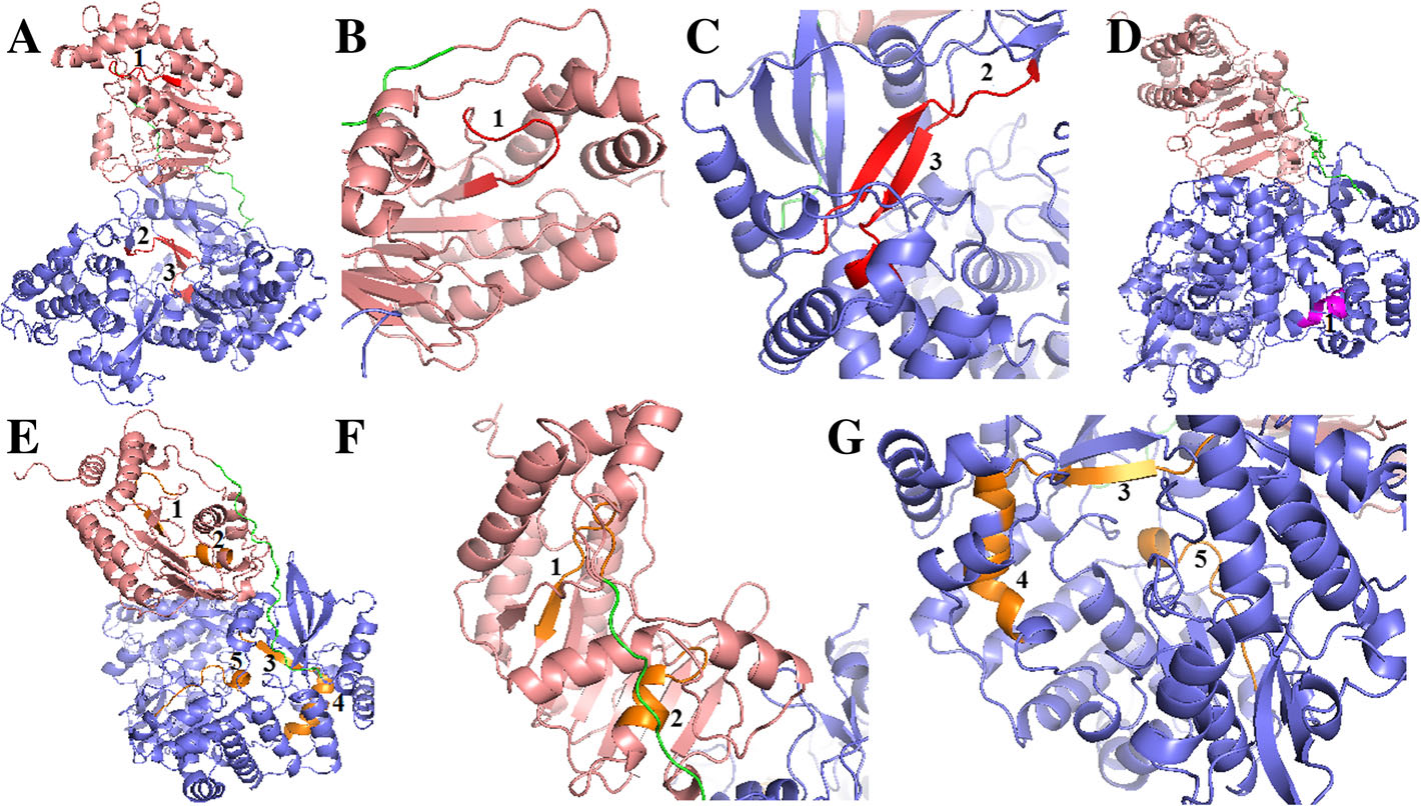
Distribution of class I HLA predicted epitopes along the ZIKV NS5 consensus 3D model. Epitopes found to be 100% conserved among ZIKV lineages and DENV serotypes were shown to be homogeneously distributed along the protein structure. Three regions containing epitopes specific for HLA-A presentation were found (**a**). One of them is located at the MTase domain and contains only one epitope (Region 1, epitope LSRNSTHEMY) (**b**). Two other regions (2 and 3) which contain together six epitopes are located at the RdRp domain (**c**). Two epitopes are located in region 2 (Region 2, epitopes CVYNMMGKR and CVYNMMGKREK) and four in region 3 (Region 3, epitopes AIWYMWLGAR, WYMWLGAR, RAIWYMWLGAR and IWYMWLGAR). One region containing only one epitope specific for HLA-B presentation is located at the RdRp domain (Region 1, epitope LEFEALGF) (**d**). Five regions containing epitopes specific for HLA-C presentation were found (**e**). Two of them are located at the MTase domain. Region 1 contains three epitopes (Region 1, epitopes SRNSTHEM, SRNSTHEMY and SRNSTHEMYW) and region 2 contains one epitope (Region 2, epitope GRGGWSYY) (**f**). Three other regions are located at the RdRp domain: region 3 contains three epitopes (Region 3, epitopes RAIWYMWL, WYMWLGAR and SRAIWYMWL), region 4 contains one epitope (Region 4, epitope LEFEALGF) and region 5 contains four epitopes (Region 5, epitopes YADDTAGWDT, YADDTAGWDTR, YADDTAGWD and ADDTAGWD) (**g**)

The structure computed for the ZIKV NS5 consensus was shown to be similar to a previous reported ZIKV NS5 structure deposited in PDB library [22]. As shown in Table 4, a high level of identity and a high TM-score were found between the consensus protein and the top ranked similar, a previous reported ZIKV NS5 protein. In addition, those 19 epitopes found to be 100% conserved among ZIKV lineages and DENV serotypes were shown to be homogeneously distributed along the protein structure (Fig. 3). Some overlapping epitopes were found in the protein and thus, their distribution is shown in regions. Three regions containing epitopes specific for HLA-A presentation were found (Fig. 3a). One of them is located at the MTase domain and contains only one epitope (Region 1, epitope LSRNSTHEMY) (Fig. 3b). Two other regions (2 and 3) which contain together six epitopes are located at the RdRp domain (Fig. 3c). Two epitopes are located in region 2 (Region 2, epitopes CVYNMMGKR and CVYNMMGKREK) and four in region 3 (Region 3, epitopes AIWYMWLGAR, WYMWLGAR, RAIWYMWLGAR and IWYMWLGAR). One region containing only one epitope specific for HLA-B presentation is located at the RdRp domain (Region 1, epitope LEFEALGF) (Fig. 3d). Five regions containing epitopes specific for HLA-C presentation were found (Fig. 3e). Two of them are located at the MTase domain. Region 1 contains three epitopes (Region 1, epitopes SRNSTHEM, SRNSTHEMY and SRNSTHEMYW) and region 2 contains one epitope (Region 2, epitope GRGGWSYY) (Fig. 3f). Three other regions are located at the RdRp domain: region 3 contains three epitopes (Region 3, epitopes RAIWYMWL, WYMWLGAR and SRAIWYMWL), region 4 contains one epitope (Region 4, epitope LEFEALGF) and region 5 contains four epitopes (Region 5, epitopes YADDTAGWDT, YADDTAGWDTR, YADDTAGWD and ADDTAGWD) (Fig. 3g).

**Table 4:** Molecular modeling results of the amino acid consensus sequence of ZIKV NS5

Rank^a^	PDB Hit^b^	TM-score^c^	RM SD^d^	IDEN^e^	Cov^f^
1	5u0bA	0.977	0.30	0.960	0.978
2	4k6mA	0.972	0.93	0.694	0.980
3	4v0qA	0.714	4.18	0.518	0.784
4	2hfzA	0.616	2.98	0.689	0.660
5	2j7uA	0.615	1.87	0.677	0.631
6	1s4fA	0.517	3.62	0.139	0.568
7	2cjqA	0.516	3.57	0.151	0.570
8	2hcsA	0.512	2.41	0.694	0.534
9	1yvxA	0.501	4.16	0.148	0.566
10	3cj0B	0.497	4.24	0.132	0.564

^a^Ranking of proteins is based on TM-score of the structural alignment between the query structure and known structures in the PDB library

^b^Digital object identifier of the top 10 proteins deposited in the PDB library which have the closest structural similarity to the ZIKV conosensus NS5;

^c^Alignment score with the most similar protein structure found in PDB library;

^d^Root-mean-square deviation of atomic positions (root-mean-square deviation, RMSD) between residues that are structurally aligned by TM-align

^e^Percentage of sequence identity in the structurally aligned region;

^f^Cov represents the coverage of the alignment by TM-align and is equal to the number of structurally aligned residues divided by length of the query protein

## Discussion

*Zika virus* (ZIKV) is a new emergent pathogen previously associated with mild infections which took off to cause microcephaly in babies and Guillain-Barré syndrome in the Americas [3–5]. *Dengue virus* (DENV1–4) [2] infects around 390 million of people per year. These two Flavivirus consists a world public health concern for which there is not specific treatment. There is not a vaccine approved for controlling ZIKV in humans. The only anti-DENV vaccine candidate approved for use in humans is based on a chimera between DENV and *Yellow fever virus* (YFV), in which envelope proteins are the only DENV-specific antigens [6–10]. Such proteins are main targets for humoral immune response. However, especially for DENV2, the vaccine formulation based on those chimeric viruses did not achieve the expected protective efficacy in phase III clinical trials. In addition, it was reported to present higher incidence of hospitalization among children [10]. Such increased hospitalization may be related to antibody dependent enhancement. Importantly, enhancement of ZIKV infection mediated by DENV-specific antibodies was observed in vitro [11–14]. Thus, the risk of inducing antibody-mediated enhancement of infection between these two major arboviruses will depend on the profile of immune response induced.

It was recently shown that protection against DENV is achieved independently of humoral immunity [19]. CD4^+^ and CD8^+^ T lymphocytes targeting non-structural protein 5 (NS5) were shown to be essential for protection. When a recombinant purified form of NS5 protein was used as a vaccine antigen, 70% of protection was achieved on a mouse model [20]. In this study, we aimed to design a vaccine antigen capable of inducing cross-protective cellular immunity against DENV and ZIKV. As the NS5 protein is the most conserved antigen between DENV and ZIKV, a ZIKV consensus amino acid sequence was shown to present a relevant number of epitopes 100% conserved among DENV serotypes and ZIKV lineages to be presented by HLA (human leucocyte antigen) alleles of different populations of the world. The designed antigen was shown to present a homogeneous distribution of epitopes and its predicted 3D model was shown to fit with the structure of a ZIKV native NS5.

A comprehensive phylogenetic analysis based on NS5 protein amino acid sequences from ZIKV and DENV isolates from different continents was carried out. It was shown that sequences from American ZIKV isolates grouped with those from isolates of the ZIKV Asian lineage. This corroborates the recent literature [23]. In addition, DENV and ZIKV NS5 proteins were shown to have diverged before their separation into different viral species and evolutionary distances could not be inferred based on phylogenetic tree. However, the consensus ZIKV NS5 protein was shown to share different numbers of epitopes with each of your homologous proteins. This result indicates that the proposed antigen could induce cross-reactive immune responses with different levels of intensity to ZIKV, DEN1, DENV2, DENV3 and DENV4.

The consensus NS5 protein was designed based on ZIKV isolates from throughout the world. Attempts to produce a ZIKV/DENV consensus resulted in a non-stable protein model (data not shown). Nevertheless, nineteen epitopes specific for presentation by class I HLA were found to be 100% conserved among DENV serotypes and ZIKV lineages. Population coverages of such epitopes with regard to different human populations in the world were shown to be relevant. Predicted vaccine coverage for populations from several tropical areas were higher than 50%. This is an important characteristic of the designed antigen considering that HLA restriction is a major challenge in vaccine development, which may significantly affect vaccine efficacy and effectiveness among human populations of different regions.

The 100% conserved epitopes were shown to be homogeneously distributed along protein domains. Although such amino acid sequences may be used in a polyepitope development project, their homogeneous distribution along the protein structure is important for using the whole antigen. As NS5 protein is a major target for CD4^+^ and CD8^+^ T lymphocytes, using the whole antigen such as we previously reported [19] would be a promising strategy in order to induce a significant immune response against the conserved epitopes. Thus, we conclude that the proposed antigen has the potential to induce protective cellular immune response to ZIKV and DENV in different human populations in the world.

## Conclusions

In the end of the analysis we conclude that NS5 consensus protein have the potential to figurate a vaccine antigen to induce cross protection to ZIKV end DENV in different human populations of the world. The next step in this process is to analyze real immune response for this antigen in mouse model and see how the immune response process will proceed.

## Additional file

Additional file 1: Table S1. –ZIKV Sequences. The sequences are named with virus species, accession number and country of isolation. Table S2 – DENV1 sequences. The sequences are named with virus species and serotype, accession number and country of isolation. Table S3 – DENV2 sequences. The sequences are named with virus species and serotype, accession number and country of isolation. Table S4 – DENV3 sequences. The sequences are named with virus species and serotype, accession number and country of isolation. Table S5 – DENV4 sequences. The sequences are named with virus species and serotype, accession number and country of isolation (DOCX 18 kb)
