# Supplementary material for: In silico design of a Zika virus non-structural protein 5 aiming vaccine protection against zika and dengue in different human populations
Source: J Biomed Sci. 2017 Nov 23;24:88. doi: 10.1186/s12929-017-0395-z (PMC5701345; doi:10.1186/s12929-017-0395-z)
Supplement: Additional file 1: Table S1. — –ZIKV Sequences. The sequences are named with virus species, accession number and country of isolation. Table S2 – DENV1 sequences. The sequences are named with virus species and serotype, accession number and country of isolation. Table S3 – DENV2 sequences. The sequences are named with virus species and serotype, accession number and country of isolation. Table S4 – DENV3 sequences. The sequences are named with virus species and serotype, accession number and country of isolation. Table S5 – DENV4 sequences. The sequences are named with virus species and serotype, accession number and country of isolation (DOCX 18 kb) [file 12929_2017_395_MOESM1_ESM.docx]

**Additional file 1: Table S1** –ZIKV Sequences. The sequences are named with virus species, accession number and country of isolation.

| **World Regions** | **Sequences** |
| --- | --- |
| North America | ZIKV_AOY08546_USA  ZIKV_AOY08537_USA  ZIKV_ARB07984_USA  ZIKV_AOY08520_USA  ZIKV_AOY08544_USA  ZIKV_AQV07165_Mexico  ZIKV_AMQ34004_Mexico  ZIKV_AMQ34003_Mexico  ZIKV_ ANN83273_Mexico  ZIKV_ANN83272_Mexico |
| Central America and Caribean | ZIKV_ANC90428_Panama  ZIKV_ANB66184_Panama  ZIKV_ARB07996_Dominican_Republic  ZIKV_ARB07953_Dominican_Republic  ZIKV_ARB07964_Honduras  ZIKV_ARB07960_Honduras  ZIKV_AQZ41956_Nicaragua  ZIKV_AQZ41955_Nicaragua  ZIKV_ASK51714_Cuba  ZIKV_ARB07976_Puerto_Rico |
| South America | ZIKV_ARB07992_Brazil  ZIKV_ARB07967_Brazil  ZIKV_ARB07962_Brazil  ZIKV_ARB07922_Brazil  ZIKV_AOY08541_Brazil  ZIKV_ARB07981_Colombia  ZIKV_AMZ03557_Colombia  ZIKV_ASU55425_Colombia  ZIKV_ASU55423_Colombia  ZIKV_ASU55416_Colombia |
| Europe | - |
| Asia | ZIKV_AOC50652_Thailand  ZIKV_ANW07475_Malaysia  ZIKV_AOC50653_Malaysia |
| Africa | ZIKV_ANW07474_Senegal  ZIKV_APO15553_Senegal  ZIKV_ANC90427_Senegal  ZIKV_AMR68906_Nigeria  ZIK_AMR68905_Uganda  ZIKV_ANW07477_Uganda  ZIKV_ABI54475_Uganda  ZIKV_ ARM59240_Uganda |
| Ocenia | - |
| Total: 41 sequences | |

**Additional file 1: Table S2** – DENV1 sequences. The sequences are named with virus species and serotype, accession number and country of isolation.

| **World Regions** | **Sequences** |
| --- | --- |
| North America | DENV1_AFJ91714_USA  DENV1_AIU47321_USA  DENV1_ACF49259_USA  DENV1_AHI43752_Mexico  DENV1_AHI43751_Mexico  DENV1_AHG23208_Mexico  DENV1_ AHG23209_Mexico  DENV1_AHG23193_Mexico  DENV1_AHI43732_Mexico  DENV1_AHG23187_Mexico |
| Central America and Caribean | DENV1_AHI43749_Puerto_Rico  DENV1_ AHI43750_Puerto_Rico  DENV1_AHI43748_Puerto_Rico  DENV1_AHI43747_Puerto_Rico  DENV1_AHC98446_Nicaragua  DENV1_AHC98445_Nicaragua  DENV1_AHC98444_Nicaragua  DENV1_AHC98443_Nicaragua  DENV1_AET43248_El_Salvador  DENV1_ALJ53459_Haiti |
| South America | DENV1_AGN94879_Brazil  DENV1_AGN94878_Brazil  DENV1_AGN94877_Brazil  DENV1_AHG23207_Venezuela  DENV1_AET43256_Venezuela  DENV1_AET43246_Venezuela  DENV1_AHI43687_Colombia  DENV_AHI43686_Colombia  DENV1_AHF50492_Argentina  DENV1_AHF50491_Argentina |
| Europe | DENV1_AIN75463_Germany |
| Asia | DENV1_AIE17470_Sri_Lanka  DENV1_AKC32653_Sri_Lanka  DENV1_AHG23212_Viet_Nam  DENV1_AHF45724_Viet_Nam  DENV1_AGT63075_Laos  DENV1_AGT63074_Laos  DENV1_ACO06148_Thailand  DENV1_AMN88556_Brunei  DENV1_AET43254_Cambodia  DENV1_ARO84721_Malaysia |
| Africa | - |
| Ocenia | DENV1_AMN88557_Australia |
| Total: 42 sequences | |

**Additional file 1: Table S3** – DENV2 sequences. The sequences are named with virus species and serotype, accession number and country of isolation.

| **World Regions** | **Sequences** |
| --- | --- |
| North America | DENV2_AHI43753_Mexico  DENV2_AHI43694_Mexico  DENV2_ AHI43692_Mexico  DENV2_AHI43693_Mexico  DENV2_AHI43691_Mexico  DENV2_AEH59346_USA  DENV2_AEH59347_USA  DENV2_AEH59342_USA  DENV2_AEH59348_USA  DENV2_AET72454_USA |
| Central America and Caribean | DENV2_AHG23138_Nicaragua  DENV2_AHG23135_Nicaragua  DENV2_BAD36759_Dominican_Republic  DENV2_BAD36760_Dominican_Republic  DENV2_APW84878_Haiti  DENV2_AOE23002_Haiti  DENV2_AHG23133_Puerto_Rico  DENV2_ACQ44488_Honduras  DENV2_AER45462_Guatemala  DENV_ AAG30730_Martinique |
| South America | DENV2_AGN94890_Brazil  DENV2_AGN94891_Brazil  DENV2_ AGN94889_Brazil  DENV2_AGN94892_Brazil  DENV2_ AHG23115_Venezuela  DENV2_AET43238_Venezuela  DENV2_AHG23153_Venezuela  DENV2_ACH99659_Colombia  DENV2_ACH61726_Colombia  DENV2_ACH61724_Colombia |
| Europe | - |
| Asia | DENV2_ARO84705_Malaysia  DENV2_ARO84704_Malaysia  DENV2_AHG23170_Cambodia  DENV2_AHG23169_Cambodia  DENV2_ACS32039_Sri_Lanka  DENV2_ACS32038_Sri_Lanka  DENV2_AHG25313_Philippines  DENV2_ASN77915_China  DENV2_BAX09288_Thailand  DENV2_AII99332_India |
| Africa | - |
| Ocenia | DENV2_ACJ04201_Papua_New_Guinea  DENV2_AIU47320_Papua_New_Guinea  DENV2_ACQ44517_Papua_New_Guinea  DENV2_AAK67712_Australia  DENV2_AAV70829_Tonga |
| Total: 45 sequences | |

**Additional file 1: Table S4** – DENV3 sequences. The sequences are named with virus species and serotype, accession number and country of isolation.

| **World Regions** | **Sequences** |
| --- | --- |
| North America | DENV3_ACQ44481_Mexico  DENV3_ACQ44480_Mexico  DENV3_ACQ44479_Mexico |
| Central America and Caribean | DENV3_AHC98458_Nicaragua  DENV3_ADU76212_Nicaragua  DENV3_ADU76210_Nicaragua  DENV3_AHC98457_Nicaragua  DENV3_AHG23233_Puerto_Rico  DENV3_AHG23231_Puerto_Rico  DENV3_AHG23230_Puerto_Rico  DENV3_AHG23221_Puerto_Rico  DENV3_AAM51538_Martinique  DENV3_AHG23270_Grenada |
| South America | DENV3_ABV03585_Brazil  DENV3_AFK83762_Brazil  DENV3_AHX22016_Peru  DENV3_AHI43684_Peru  DENV3_AHG23252_Venezuela  DENV3_ACW82877_Venezuela  DENV3_AFK83764_Paraguay  DENV3_AFK83763_Paraguay  DENV3_ ADA60766_Colombia  DENV3_ ACQ44496_Ecuador |
| Europe | - |
| Asia | DENV3_AAX19004_Thailand  DENV3_AAW66608_Thailand  DENV3_ASN77913_China  DENV3_ALJ02589_China  DENV3_AFI55000_Sri_Lanka  DENV3_AAM51537_Sri_Lanka  DENV3_AHG23229_Cambodia  DENV3_AAT69740_Indonesia  DENV3_AHG23242_India  DENV3_AHG23225_Viet_Nam |
| Africa | DENV3_ ACQ44384_Mozambique |
| Ocenia | DENV3_AFN80339_Australia  DENV3_AFN80338_Australia |
| Total: 36 sequences | |

**Additional file 1: Table S5** – DENV4 sequences. The sequences are named with virus species and serotype, accession number and country of isolation.

| **World Regions** | **Sequences** |
| --- | --- |
| North America | - |
| Central America and Caribean | DENV4_ACS32019_Puerto_Rico  DENV4_ACS32037_Puerto_Rico  DENV4_ACS32018_Puerto_Rico  DENV4_ACS32017_Puerto_Rico  DENV4_ACS32016_Puerto_Rico  DENV4_ACS32014_Puerto_Rico  DENV4_ACS32015_Puerto_Rico  DENV4_ACS32013_Puerto_Rico  DENV_ALJ53458_Haiti  DENV4_AEX09560_Haiti |
| South America | DENV4_AEW50183_Brazil  DENV4_ANK35835_Brazil  DENV_AKQ00037_Brazil  DENV_AKQ00033_Brazil  DENV4_ACW83013_Colombia  DENV4_ACW83012_Colomba  DENV4_ACW83011_Colombia  DENV4_AET43237_Venezuela  DENV4_AET43240_Venezuela  DENV4_ACW82933_Venezuela |
| Europe | - |
| Asia | DENV4_AHG23274_Cambodia  DENV4_AHG23290_Cambodia  DENV4_AHG23289_Cambodia  DENV4_AHG23288_Cambodia  DENV4_AHG23275_Cambodia  DENV4_ACW82884_Philippines  DENV4_AEX09561_India  DENV4_AEX09558_Malaysia  DENV4_AEX09557_Malaysia  DENV4_AHN50410_Sri_Lanka |
| Africa | - |
| Ocenia | - |
| Total: 30 sequences | |

Total geral: 194 sequencias
